# Linkage mapping and association analysis to identify a reliable QTL for stigma exsertion rate in rice

**DOI:** 10.3389/fpls.2022.982240

**Published:** 2022-08-23

**Authors:** Yi Liu, Dong Fu, Deyan Kong, Xiaosong Ma, Anning Zhang, Feiming Wang, Lei Wang, Hui Xia, Guolan Liu, Xinqiao Yu, Lijun Luo

**Affiliations:** ^1^Shanghai Agrobiological Gene Center, Shanghai, China; ^2^Key Laboratory of Grain Crop Genetic Resources Evaluation and Utilization, Ministry of Agriculture and Rural Affairs, Shanghai, China; ^3^State Key Laboratory of Biocatalysis and Enzyme Engineering, School of Life Sciences, Hubei University, Wuhan, China

**Keywords:** quantitative trait locus (QTL), genome-wide association study (GWAS), transcriptome analysis, stigma exsertion rate (SER), rice

## Abstract

The commercialization of hybrid rice has greatly contributed to the increase in rice yield, with the improvement of its seed production capacity having played an important role. The stigma exsertion rate (SER) is a key factor for improving the outcrossing of the sterile line and the hybrid rice seed production. We used the Zhenshan 97B × IRAT109 recombinant inbred population comprising 163 lines and a natural population of 138 accessions to decipher the genetic foundation of SER over 2 years in three environments. Additionally, we detected eight QTLs for SER on chromosomes 1, 2, and 8 via linkage mapping. We also identified seven and 19 significant associations for SER using genome-wide association study in 2016 and 2017, respectively. Interestingly, we located two lead SNPs (sf0803343504 and sf083344610) on chromosome 8 in the *qTSE8* QTL region that were significantly associated with total SER. After transcriptomic analysis, quantitative real-time PCR, and haplotype analysis, we found 13 genes within this reliable region as important candidate genes. Our study results will be beneficial to molecular marker-assisted selection of rice lines with high outcrossing rate, thereby improving the efficiency of hybrid seed production.

## Introduction

Over half the global population heavily relies on rice (*Oryza sativa* L.) as a primary source of nutrition, with the demand set to increase with the growing population. To match the current population growth rate, rice production needs to increase by at least 40 percent by 2030 (Khush, 2005). Therefore, hybrid rice was developed, which showed 10–20% greater yield than most of the conventional rice cultivars. Improving the hybrid rice yield may be one viable method of addressing the potential food scarcity associated with an ever-increasing global population (Cheng et al., 2007; Spielman et al., 2013). Unfortunately, since rice is self-pollinated, achieving sufficient outcrossing to produce hybrid seeds can be difficult (Kato and Nimai, 1987), with poor seed yield being a major hybrid rice production constraint. Therefore, the challenge is to improve the hybrid rice seed production by enhancing rice outcrossing success (Virmani, 1994; Marathi and Jena, 2014). Several flower traits have been linked to hybrid seed production efficiency, anther pollen density and quality, flowering behavior (e.g., anthesis interval and heading date), and stigma size and exsertion of sterile males (Virmani et al., 1982). Exserted stigmas are specifically less affected by glume shielding and remain viable for several days, thereby increasing the likelihood of trapping exotic pollen and consequently improving rice cross-pollination (Sidharthan et al., 2007). Thus, the stigma exsertion rate (SER) is singularly crucial for the enhancement of hybrid rice seed production.

With the continuous advancement of molecular marker technologies over the past several years, researchers have successfully mapped multiple SER-related QTLs in rice utilizing various types of segregating populations, including chromosome segment substitution lines (Rahman et al., 2017; Zhang et al., 2018; Tan et al., 2020, 2021), doubled haploid lines (Hittalmani et al., 2002; Li et al., 2003), F_2_ populations (Xiong et al., 1999; Yue et al., 2009; Li et al., 2010, 2017; Xu et al., 2019), backcrossing populations (Li et al., 2001; Miyata et al., 2007), and recombinant inbred lines (RILs) (Uga et al., 2003; Yamamoto et al., 2003; Li et al., 2014). Several studies have identified one particular QTL that co-localized with the *GS3* gene, which controls grain size, stigma length, and stigma exsertion (Miyata et al., 2007; Takano-Kai et al., 2011). Rahman et al. (2017) and Zhang et al. (2018) dissected two major QTLs (*qSE11* and *qSE7*), demarcating them into precise regions on chromosomes 11 and 7, respectively. Another QTL (*qSER7*) was fine-mapped to a ~28 kb region on chromosome 7 (Liu et al., 2019). Therefore, these efforts have shown that SER is a particularly complex trait that is strongly affected by the environment.

Although several QTLs have been identified, only a few have either been cloned or fine-mapped. A considerable limitation of the traditional linkage-based QTL mapping is that it allows the study of only two alleles at any particular locus (Dang et al., 2016). Conversely, genome-wide association studies (GWAS), which utilize the linkage disequilibrium (LD) between phenotypes and genotypes, were promising for localizing QTLs controlling complex traits. Another technology which has vastly aided the advancement of GWAS is next-generation high-throughput DNA sequencing (Brachi et al., 2010; Huang et al., 2013). Recently, GWAS-based QTL mapping has successfully identified SER-associated loci (Yan et al., 2009; Huang et al., 2012; Guo et al., 2017). For example, Zhou et al. (2017) used GWAS to identify over 20 stigma exsertion-associated genomic loci, with three of them being co-localized with the three primary genes controlling grain size *GS3, GW5*, and *GW2*.

In the present study, we sought to map the rice SER QTLs via linkage analysis that utilizes an RIL population and association analysis by using a natural population. Using these two methods under different environments, we identified a reliable SER QTL on chromosome 8. Based on transcriptomic analysis, quantitative real-time PCR and haplotype analysis, we obtained 13 candidate genes within the identified QTL region. The results will helpful both fine mapping and gene cloning of QTL for SER and also support molecular marker-assisted selection of rice lines with high outcrossing.

## Materials and methods

### Plant materials and field experiments

Two collections of rice were utilized for this study (Collection 1 and 2). Collection 1, which was used for traditional linkage-based QTL mapping, encompassed 163 F_10_ RILs developed from Zhenshan 97B (high SER *indica* rice) and IRAT109 (low SER *japonica* rice) (Zou et al., 2005; Liu et al., 2008; Lou et al., 2015). Collection 2, which was used for GWAS analysis, encompassed 138 *indica* subpopulation accessions of the Chinese rice germplasm mini-core collection (Wu et al., 2015; Ma et al., 2016).

Both rice collections were field-grown using conventional rice cultivation methods and staged sowing at the Shanghai Agrobiological Gene Center field stations in Hainan and Shanghai, China. The field sites were arranged in triplicates based on a randomized block design. Rice was sown seven plants per row in five rows, with a spacing of 18 x 16 cm between and within rows. Collection 1 was analyzed during the summer of 2016 in Shanghai, while in spring of 2017 and 2018 in Hainan. Collection 2 was analyzed during the summer of 2016 in Shanghai and spring 2017 in Hainan.

### Trait evaluation

Eight panicles from each rice line were sampled 5–7 days post spikelet flowering. For analysis, we subdivided SER into three separate traits: total stigma exsertion rate (TSE), single stigma exsertion rate (SSE), and dual stigma exsertion rate (DSE). The TSE, SSE, and DSE were determined as a percentage of rice spikelets displaying these traits.

### Genotyping

The method of Zou et al. (2005) was followed to characterize the Collection 1 genotypes by using 213 simple sequence repeats (SSR) as markers. Collection 2 was subjected to whole-genome resequencing utilizing an Illumina Solexa Hiseq 2000 sequencing system. All raw sequences can be found online: https://www.ncbi.nlm.nih.gov/Traces/index.html?view=run_browser&acc=SRR1239601&display=metadata and http://www.ncbi.nlm.nih.gov/bioproject/PRJNA260762. Clean reads were used to identify single nucleotide polymorphisms (SNPs) using a combination of BCFtools (Li et al., 2009), SAMtools, and BWA (Li and Durbin, 2009). In total, 1,019,883 SNPs were identified. The SNP identification accuracy was evaluated by subjecting 24 accessions to RiceSNP50, a whole-genome, high-density SNP array (Chen H. et al., 2014). Details regarding the specific methods requires for the processing of the genomic data can be found in Chen W. et al. (2014).

### Linkage analysis

Phenotypic information was analyzed with SPSS ver. 19. The MAPMAKER/EXP 3.0 was used to construct linkage maps of the different genotypes (Lander et al., 1987). A mixed-model-based composite interval mapping (MCIM) method was used to perform QTL analysis by applying QTLNetwork ver. 2.0 (Yang et al., 2007, 2008). Hypotheses were tested using the Henderson method III F-statistic, with primary QTLs being declared at F > 6.4. By using a significance level of *p* < 0.05, the threshold was computed using a 1,000-shuffle permutation test (Churchill and Doerge, 1994).

### Association analysis

A compressed mixed linear model method (Zhang et al., 2010) was used to conduct the GWAS, utilizing the R package “Genomic Association and Prediction Integrated Tool (GAPIT)” (Lipka et al., 2012). A minimum allelic frequency (MAF) of 5% was used for all SNPs. The reference genome used was Nipponbare (MSU6.0) (http://rice.plantbiology.msu.edu/). The local LD-based interval of reliable significant SNPs was considered the candidate region, where the LD between nearly SNPs and lead SNP (with the lowest *p*-value) reduced to r^2^ = 0.6 (Yano et al., 2016).

### Transcriptome analysis

During our previous study (Xia et al., 2020), Zhenshan 97B and IRAT109 were planted as three biological replicates in 2014 at the Baihe Experimental Station in Shanghai, China. Three top leaf samples from three individuals of each replicate at the pre-heading stage were collected on August 5th, 2014, and subsequently stored in liquid nitrogen prior to RNA sequencing. The Illumina Hiseq 2500 at Shanghai Majorbio Biopharm Technology Co., Ltd. (Shanghai, China) was used for RNA sequencing. The PureLink^®^ Plant RNA Reagent (Thermo Fisher Scientific, MA, USA) was utilized to extract total RNA. Using SeqPrep (https://github.com/najoshi/sickle), the single reads were created using overlapping paired reads, with the adaptors being stripped. The library was constructed according to the TruSeq^®^ RNA Sample Preparation v2 Guide (Illumina) using qualified RNA. Cufflinks and Tophat were utilized to map clean data to the Nipponbare (MSU6.0) reference genome (http://rice.plantbiology.msu.edu/), allowing two or less alignment mismatches (Trapnell et al., 2012). Raw sequences can be found online: https://www.ncbi.nlm.nih.gov/bioproject/PRJNA609211. The Fragment Per Kilobase of exon per Million fragments mapped (FPKM) method was utilized to determine the gene expression levels by using the Cuffdiff software (Trapnell et al., 2012).

### Quantitative real-time PCR analysis

Total RNA was extracted from young panicles (Stage In7 to Stage In8, with panicle lengths of 5–100 mm) of Zhenshan 97B and IRAT109 at the pre-heading stage using TRNzol-A+ Total RNA Reagent (TIANGEN, Beijing, China). The qRT-PCR method was described by a previous study (Liu et al., 2019). The primers were listed in Supplementary Table 1.

### Haplotype analysis

Haplotype analysis was performed on all genes in the reliable candidate region. The SNPs within the 2.0 kb promoter region along with the non-synonymous SNPs in the exon regions of all genes in the interval were used to perform haplotype analysis using the R software.

## Results

### Phenotypic analysis

The basic SER traits of Collection 1 (RILs) and Collection 2 across 3 years are shown in Table 1. For Collection 1, both the single and dual SERs of Zhenshan 97B were superior to those of IRAT109. Because of the nature of Collection 2, it displayed a wider range and lower coefficient of variation for all SER traits compared to Collection 1. The TSE, SSE, and DSE exhibited environmentally-dependent correlations with each other (Table 2 and Supplementary Table 2). During the spring of 2018 in Hainan, the SSE (SSE2018) and TSE (TSE2018) exhibited the strongest phenotypic correlation (r = 0.987), followed by DSE2018 and TSE2018 (r = 0.794) and SSE2018 and DSE2018 (r = 0.684). The results were similar in the other two environments. Overall, the lines with the greatest SSE were also likely to exhibit increased DSE and TSE.

**Table 1:** Phenotypic information regarding the stigma exsertion rate of the parents and two collections across 3 years.

**Trait**	**Year/Site**	**Parent**		**Collection 1**				**Collection 2**			
		**Zhenshan 97B**	**IRAT109**	**Mean**	**SD**	**Range**	**CV**	**Mean**	**SD**	**Range**	**CV**
SSE/%	2016 Shanghai	21.2	15.4	6.1	6.8	0–42.1	1.1	20.0	16.1	0.5–51.4	0.8
	2017 Hainan	29.9	16.3	11.0	9.3	0.1–36.1	0.9	19.3	15.9	0.5–51.3	0.8
	2018 Hainan	26.8	14.2	9.0	8.5	0–38.1	0.9				
DSE/%	2016 Shanghai	3.5	0.3	0.4	1.3	0–13.3	3.8	6.9	10.3	0–39.7	1.5
	2017 Hainan	4.7	0.8	1.5	3.0	0–22.5	2.0	5.9	9.1	0–39.8	1.5
	2018 Hainan	4.0	0.5	1.0	2.3	0–23.9	2.3				
TSE/%	2016 Shanghai	24.7	15.7	6.4	7.8	0–55.4	1.2	26.8	25.4	0.5–80.5	0.9
	2017 Hainan	34.6	17.1	12.5	11.4	0.1–48.1	0.9	25.2	22.1	0.6–85.6	0.9
	2018 Hainan	30.8	14.7	9.9	10.2	0–53.4	1.0				

CV, coefficient of variation; SD, standard deviation; TSE, total stigma exsertion rate; SSE, single stigma exsertion rate; DSE, dual stigma exsertion rate.

**Table 2:** Pearson correlation coefficients between the traits of Collection 1 over 3 years.

	**SSE2016**	**DSE2016**	**TSE2016**	**SSE2017**	**DSE2017**	**TSE2017**	**SSE2018**	**DSE2018**	**TSE2018**
SSE2016	1								
DSE2016	0.729**	1							
TSE2016	0.979**	0.797**	1						
SSE2017	0.528**	0.197*	0.511**	1					
DSE2017	0.358**	0.267**	0.373**	0.666**	1				
TSE2017	0.514**	0.224**	0.501**	0.980**	0.797**	1			
SSE2018	0.393**	0.122	0.372**	0.643**	0.545**	0.667**	1		
DSE2018	0.161	0.058	0.151	0.355**	0.682**	0.470**	0.684**	1	
TSE2018	0.362**	0.115	0.343**	0.615**	0.613**	0.662**	0.987**	0.794**	1

TSE, total stigma exsertion rate; SSE, single stigma exsertion rate; DSE, dual stigma exsertion rate; ^**^ and ^*^, 2-tailed significance at p < 0.01 and < 0.05, respectively.

### Linkage-based QTL mapping

Previously, a set of 213 SSRs was utilized to create a linkage map of the RIL population (Zou et al., 2005), which was utilized here, along with phenotypic data, for SER QTL mapping (Table 3). We identified eight SER QTLs distributed on chromosomes 1, 2, and 8, with the QTL F-values ranging from 7.3 to 12.2 that explained a range of the phenotypic variation of 4.02–10.27%. Among these, we discovered three QTLs for TSE on chromosomes 1, 2, and 8 (Supplementary Figure 1). The QTL-*qTSE8*, flanked by RM38 and RM25, had the largest additive effect and explained 10.01% of the phenotypic variation. Both alleles, i.e., *qTSE1* and *qTSE8* were inherited from Zhenshan 97B, whereas the alleles *qTSE2* was derived from IRAT109. For SSE, we found three QTLs on chromosomes 1, 2 and 8 (Supplementary Figure 2). The QTL-*qSSE1*, flanked by RM220 and RM490, explained 10.27% of the variation in phenotypes. *qSSE1* and *qSSE8* indicated a positive additive effect, whereas *qSSE2* demonstrated a negative effect. For DSE, two QTLs (*qDSE1* and *qDSE8*) derived from the parent Zhenshan 97B, were discovered on chromosomes 1 and 8 (Supplementary Figure 3). The ratio of environmental variation to phenotypic variation [V(E)/V(P)] was 24.67%, while the ratio of variation due to environment x genotype and phenotypic variation [V(GE)/V(P)] was 4.40%, thereby suggesting that environmental factors only had a minor effect.

**Table 3:** Putative stigma exsertion rate QTLs detected by linkage mapping in Collection 1.

**Traits**	**QTL**	**Chr**.	**Interval**	**Physical position (bp)**	**F-Value**	**A (%)**	**PVE (%)**	**AE1**	***P*-Value**	**AE2**	***P*-Value**	**AE3**	***P*-Value**
SSE	*qSSE1*	1	RM220–RM490	4,425,496–6,677,249	12.2	3.01	10.27	−0.42	0.31	0.27	0.51	0.15	0.70
	*qSSE2*	2	RM6–RM240	29,585,840–31,503,125	7.3	−1.66	4.39	0.00	0.99	0.00	0.99	0.00	1.00
	*qSSE8*	8	RM25–RM544	4,378,457–5,109,223	11.5	1.98	9.01	−0.75	0.14	1.04	0.04	−0.29	0.54
DSE	*qDSE1*	1	RM490–RM259	6,677249–7,446,813	7.3	0.48	4.02	−0.17	0.25	0.20	0.18	−0.02	0.86
	*qDSE8*	8	RM152–RM52	684,095–24,757,839	8.7	0.45	4.30	−0.13	0.33	0.10	0.42	0.02	0.84
TSE	*qTSE1*	1	RM220–RM490	4,425,496–6,677,249	11.6	2.84	9.54	0.00	0.99	0.00	0.99	0.00	0.99
	*qTSE2*	2	RM6–RM240	29,585,840–31,503,125	7.9	−2.15	4.53	0.00	0.99	0.00	0.99	0.00	1.00
	*qTSE8*	8	RM38–RM25	2,115,840–4,378,457	11.6	3.07	10.01	−1.28	0.08	1.05	0.15	0.21	0.75

Chr, chromosome position of candidate QTL; F-value, F value of the putative QTLs obtained by F-statistic; A, estimated additive effect of the QTLs, a positive A-value implies that the P1 parent (Zhenshan 97B) takes a positive value for the additive effect and a negative A-value means that the P2 parent (IRAT109) takes a positive value for the additive effect; PVE, the phenotypic variance explained by each QTL; p-value, p-value of the predicted QTL effect; AE1, AE2, and AE3: are the predicted additive effects from the environmental interaction effect in the experiments of 2016 Shanghai, 2017 Hainan, and 2018 Hainan, respectively.

### LD-based association mapping

**Figure 1:**
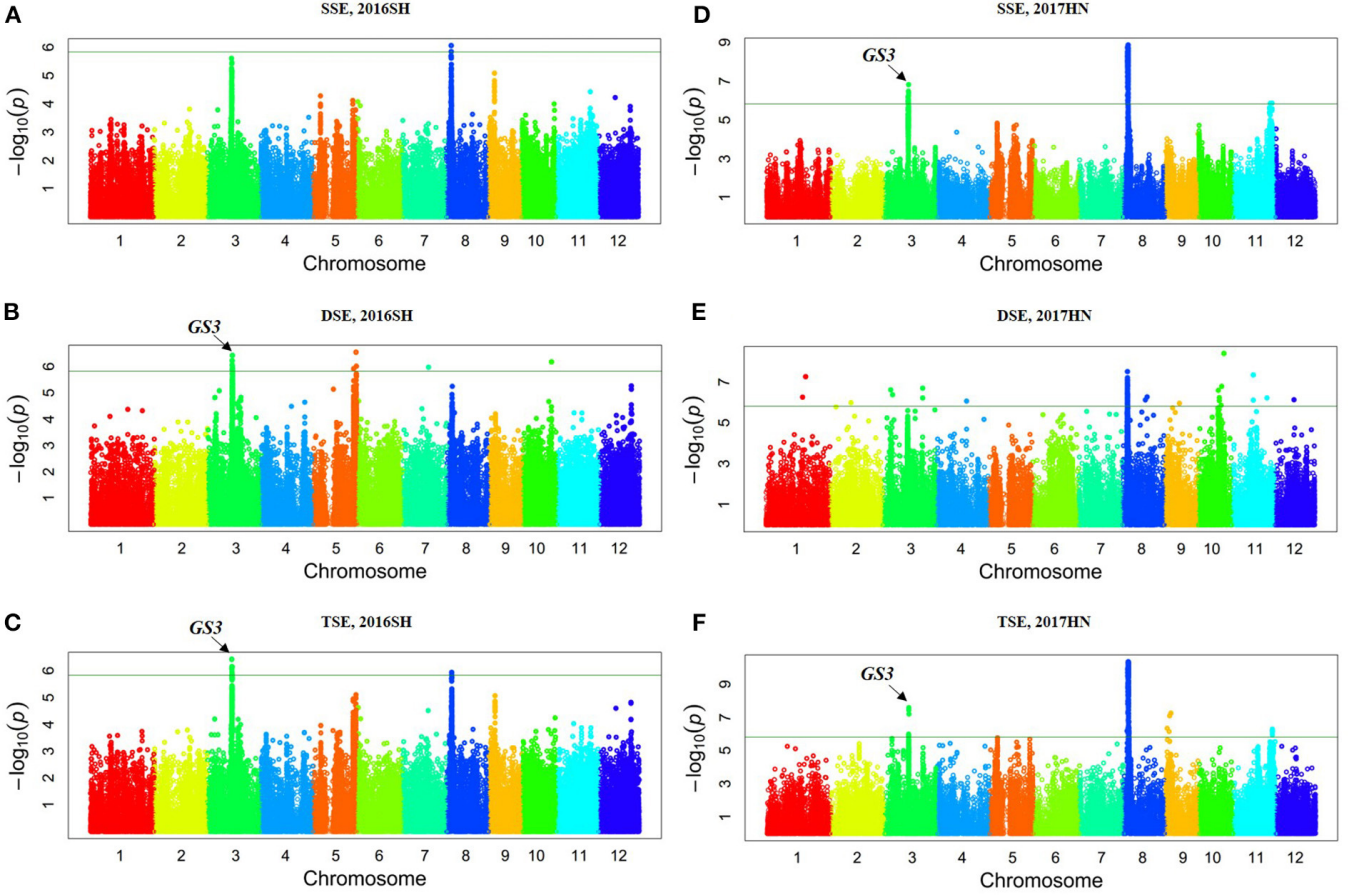
Manhattan plots of genome-wide association mapping for the stigma exsertion rate in Collection 2. 2016SH: 2016 Shanghai **(A–C)**; 2017HN: 2017 Hainan **(D–F)**; TSE, total stigma exsertion rate; SSE, single stigma exsertion rate; DSE, dual stigma exsertion rate.

Genotyping of Collection 2 yielded 1,019,883 SNP markers, with a mean distribution of 2.7 SNPs per kb. The majority of these were located in the intergeneric regions (69.6%), with a minority located within coding sequences (13.2%). Utilizing the phenotypic information of 138 Collection 2 accessions, a GWAS was performed using GAPIT (MAF > 5%), with -log(P) ≥ 6.0 as the threshold at a significance level of *p* < 0.01. For SER (2016SH), we found two, one, and four significant associations for TSE, SSE, and DSE, respectively (Figures 1A–C). We identified a particularly strong signal at the same 3,343,504 bp locus on chromosome 8 for both SSE and TSE. Also, we found the associated SNP sf0316682766 on chromosome 3 near *GS3* for both TSE and DSE. We detected other TSE-associated loci on chromosomes 3 and 4, and also other DSE-associated loci on chromosomes 3, 5, 7, and 10. In total, we discovered nineteen significant loci, including three for SSE, twelve for DSE, and four for TSE in 2017HN (Figures 1D–F). The associated loci were distributed on chromosomes 1, 2, 3, 4, 8, 9, 10, 11, and 12, respectively. Two lead SNPs (sf0316871583 and sf0316777036) located on chromosome 3 were associated with SSE and TSE, respectively. Furthermore, the three lead SNPs (sf0803464142, sf0803434218, and sf0803344610) located on chromosome 8 were associated with SSE, DSE and TSE, respectively (Table 4).

**Table 4:** Summary of GWAS loci for stigma exsertion rate in Collection 2.

**Traits**	**Chromosome**	**Lead SNP**	**Peak position (bp)**	**Peak value**	**Minor allele frequency**	**Known gene**	**Distance (kb)**	**Previous study**
SSE2015	8	sf0803343504	3,343,504	8.71E-07	0.32			
SSE2016	3	sf0316871583	16,871,583	8.55E-07	0.38	*GS3 (Os03g0407400)*	14.2	Takano-Kai et al., 2011
	8	sf0803464142	3,464,142	1.36E-09	0.27			
	11	sf1126538122	26,538,122	1.34E-06	0.2			
DSE2015	3	sf0316682766	16,682,766	5.86E-07	0.39	*GS3 (Os03g0407400)*	4.7	Takano-Kai et al., 2011
	5	sf0528961670	28,961,670	2.81E-07	0.17			Deng et al., 2011
	7	sf0717085909	17,085,909	1.05E-06	0.07			
	10	sf1019356542	19,356,542	6.57E-07	0.06			Li et al., 2014; Guo et al., 2017
DSE2016	1	sf0126895418	6,895,418	5.45E-08	0.05			Li et al., 2010
	2	sf0214392383	14,392,383	1.03E-06	0.08			
	3	sf0305361988	5,361,988	2.39E-07	0.07			
	3	sf0327022422	27,022,422	1.99E-07	0.05			
	4	sf0420363387	20,363,387	8.55E-07	0.06			
	8	sf0803434218	3,434,218	3.08E-08	0.16			
	8	sf0816735944	16,735,944	5.34E-07	0.05			
	9	sf0910197761	10,197,761	1.10E-06	0.07			
	10	sf1017390934	17,390,934	3.97E-09	0.11			Yu et al., 2006
	11	sf1114176991	14,176,991	7.70E-07	0.1			
	11	sf1123454256	23,454,256	6.00E-07	0.07			
	12	sf1213234113	13,234,113	7.37E-07	0.5			
TSE2015	3	sf0316682766	16,682,766	3.74E-07	0.39	*GS3 (Os03g0407400)*	4.7	Takano-Kai et al., 2011
	8	sf0803343504	3,343,504	1.44E-06	0.32			
TSE2016	3	sf0316777036	16,777,036	1.01E-06	0.49	*GS3 (Os03g0407400)*	4.8	Takano-Kai et al., 2011
	8	sf0803344610	3,344,610	4.50E-11	0.22			
	9	sf0902969755	2,969,755	7.97E-08	0.06			
	11	sf1126533484	26,533,484	5.11E-07	0.29			

SSE, single stigma exsertion rate; DSE, dual stigma exsertion rate; TSE, total stigma exsertion rate.

### Comparison of results from QTL and GWAS

According to QTL analysis, we found that *qTSE8* on chromosome 8 (RM38-RM25, Chr8: 2,115,840–4,378,457) was associated with TSE (Table 3). Similarly, according to the GWAS analysis, the two peak position SNPs (sf0803343504 and sf083344610) on chromosome 8 were associated with TSE in 2016 and 2017 (Figure 1 and Table 4). Notably, we identified and found that these two associated loci were located in the QTL *qTSE8* region via linkage-based mapping.

### Identification of candidate genes

**Figure 2:**
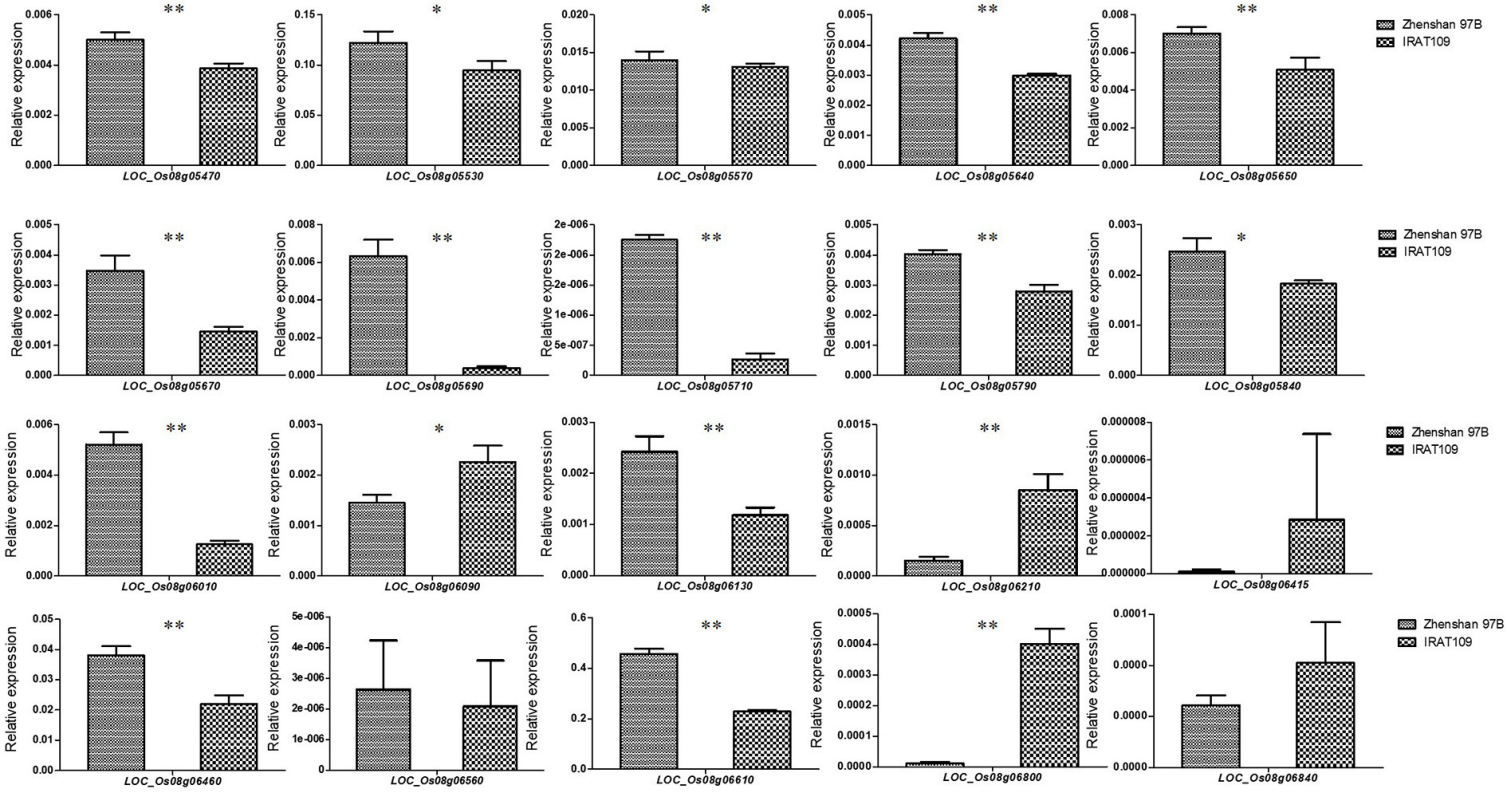
The qRT-PCR analysis of the expression of candidate genes between Zhenshan 97B and IRAT109. *p < 0.05; **p < 0.01; Student's *t*-test. Values are the mean ± SD with three biological replicates.

**Figure 3:**
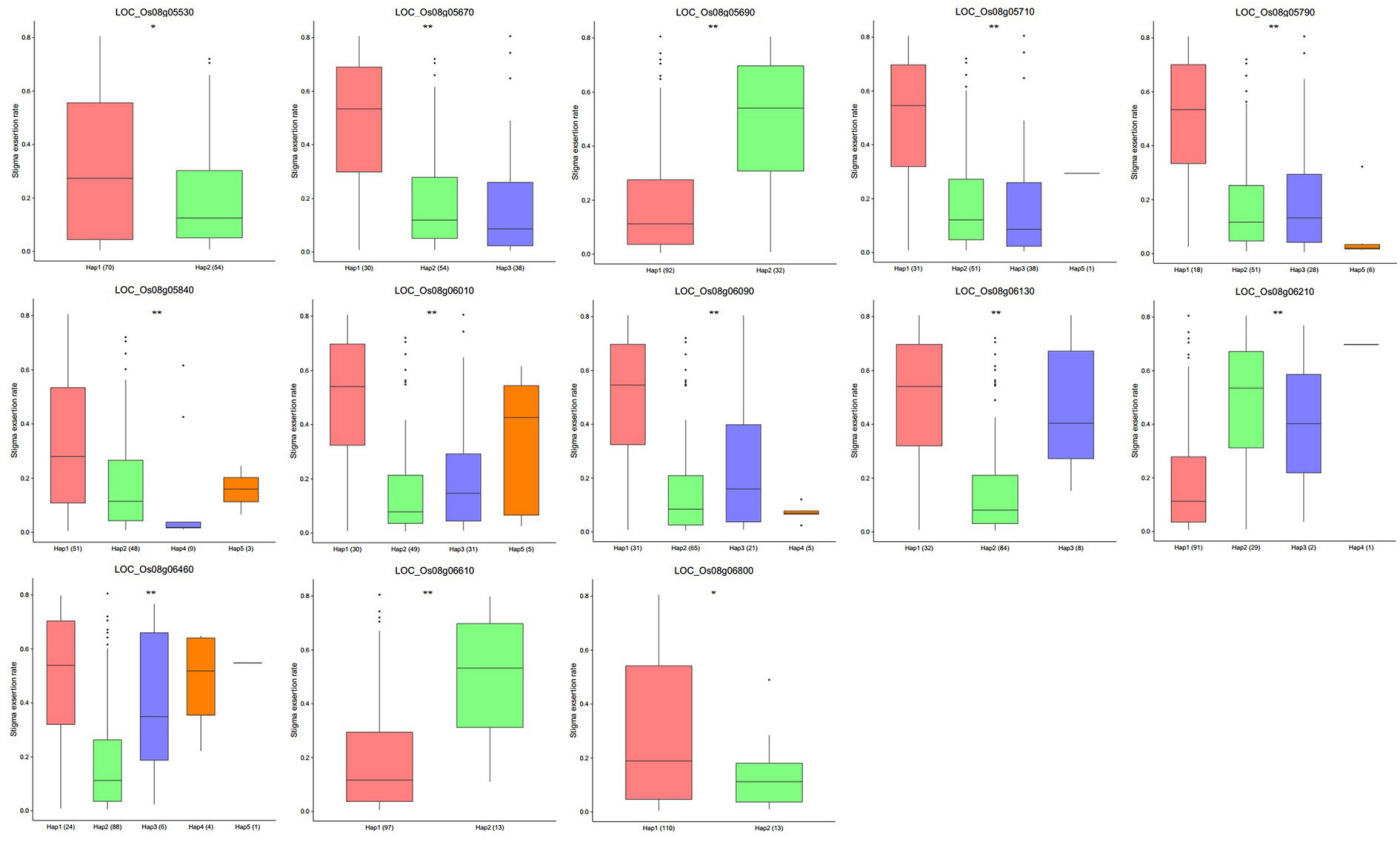
Haplotype analysis of the candidate genes. *p < 0.05; **p < 0.01; Student's *t*-test.

According to the colocalization results, *qTSE8* can be considered as a reliable locus. We reduced the candidate interval of *qTSE8* on chromosome 8 to 900 kb (2.90–3.80 Mb, r2 of LD > 0.6), and found there were 20 genes within this region, showing significantly different gene expression levels between Zhenshan 97B and IRAT109 based the transcriptome data (Supplementary Table 3). Furthermore, we evaluated these twenty genes via qRT-PCR in the young panicle tissues of the two parents to verify the varying expression levels of the candidate genes. Besides *LOC_Os08g06415, LOC_Os08g06560*, and *LOC_Os08g06840*, the expression levels of the other 17 genes showed significant or extremely significant differences between Zhenshan 97B and IRAT109 (Figure 2). According to the rice genome annotation database, these 17 candidate genes, included 13 functionally annotated genes, two genes encoding a conserved hypothetical protein, one gene encoding a protein with unknown functions, and one gene encoding retrotransposon protein (Table 5). The FPKM and qRT-PCR results of the 17 genes have the same trend (Figure 2 and Supplementary Table 3). The expression of most candidate genes in Zhenshan 97B was significantly higher than that in IRAT109. We conducted haplotype analysis on both the SNPs lying within the promoter region and the non-synonymous SNPs in the exon region of those 17 candidate genes. Among them, 13 genes were associated with significant differences in the SER among the different haplotypes (Figure 3 and Supplementary Table 4). The other 4 genes (*LOC_Os08g05470, LOC_Os08g05570, LOC_Os08g05640*, and *LOC_Os08g05650*) exhibited no significant differences regarding the SER between different haplotypes. Two haplotypes were found for *LOC_Os08g05530, LOC_Os08g05690, LOC_Os08g06610*, and *LOC_Os08g06800*. Three haplotypes were found for *LOC_Os08g05670* and *LOC_Os08g06130*. The other genes were divided into four or five haplotypes. Combined with these analysis results, we finally obtained 13 rice SER-related genes in the candidate interval, which should be focused on in subsequent research.

**Table 5:** Annotation information of candidate genes identified via linkage analysis and GWAS.

**Candidate gene**	**Start**	**End**	**Comment information**
*LOC_Os08g05470*	2,909,609	2,912,538	Conserved hypothetical protein
*LOC_Os08g05530*	2,965,358	2,968,015	LSM domain containing protein, expressed
*LOC_Os08g05570*	2,977,391	2,982,700	Monodehydroascorbate reductase, putative, expressed
*LOC_Os08g05640*	3,013,718	3,016,330	Protein of unknown function DUF1336 domain containing protein
*LOC_Os08g05650*	3,017,356	3,022,047	Diacylglycerol kinase, putative, expressed
*LOC_Os08g05670*	3,031,282	3,038,465	Armadillo-like helical domain containing protein
*LOC_Os08g05690*	3,050,168	3,056,073	ABC transporter, ATP-binding protein, putative, expressed
*LOC_Os08g05710*	3,061,866	3,067,858	ABC transporter, ATP-binding protein
*LOC_Os08g05790*	3,101,979	3,105,447	O-methyltransferase, family 3 protein
*LOC_Os08g05840*	3,136,744	3,142,891	DNA topoisomerase 1, putative, expressed
*LOC_Os08g06010*	3,281,097	3,284,500	Transporter, major facilitator family, putative, expressed
*LOC_Os08g06090*	3,326,351	3,327,611	Zinc finger, RING-type domain containing protein
*LOC_Os08g06130*	3,382,471	3,387,009	Conserved hypothetical protein
*LOC_Os08g06210*	3,425,736	3,428,074	Expressed protein
*LOC_Os08g06460*	3,622,704	3,624,999	dnaJ domain containing protein, expressed
*LOC_Os08g06610*	3,726,902	3,727,461	mps one binder kinase activator-like 1A
*LOC_Os08g06800*	3,792,098	3,795,538	Retrotransposon protein, putative, unclassified, expressed

## Discussion

One crucial trait for the improvement of hybrid rice seed production is SER, a trait linked to the female parent. Research on SER indicates that stigma exsertion is not only regulated by QTLs, but also potentially affected by environmental conditions. Although multiple SER-associated QTLs have been characterized to date, few were placed within a 500 kb interval on the chromosomes (Tan et al., 2021). Unfortunately, traditional QTL mapping of bi-parental crosses is imprecise, and it can only identify the better alleles between the two parents. QTL mapping with GWAS has shown promise for precisely localizing QTLs. GWAS makes the fine-scale mapping of QTLs possible because it utilizes linkage disequilibrium (LD) to explore the interconnection between the phenotypic variation and genotype (Mackay et al., 2009; Guo et al., 2017). The two mapping strategies are complementary to each other in terms of accuracy and breadth of QTL location, information provided, statistical analysis methods, etc. Therefore, combining the two mapping strategies can greatly improve the study of complex quantitative traits (Lou et al., 2015).

In this study, we combined both LD-based and linkage-based mapping to understand the genetic foundation of SER in rice. The QTLs detected for SSE also matched TSE, and we also found that these two traits were highly correlated. Using the RIL population grown in different environments, we detected eight SER QTLs. Three QTLs, namely, *qSSE1, qDSE1*, and *qTSE1* showed chromosomal regions overlapping with those previously described in Li et al. (2010), Li et al. (2014), and Rahman et al. (2017), respectively. The QTLs *qSSE8* and *qTSE8* were identified adjacent to the chromosomal regions *qSPES-8* and *qPES-8* identified previously (Deng et al., 2010). Taken together, these findings confirm the accuracy of our identified QTLs.

We detected seven and 19 significant loci through LD-based association analysis in 2016SH and 2017HN, respectively. Furthermore, we detected the same significant SER-associated peak on chromosome 3 near *GS3* in 2 years (*p* < 10^−6^). Previous research indicated that the *GS3* gene controled stigma exsertion (Takano-Kai et al., 2011; Zhou et al., 2017). Our study confirmed that the grain length gene *GS3* indeed affected SER, thereby indicating that our GWAS results were reliable. We found the significant DSE loci on chromosomes 1, 5, and 10 to be located within regions of the genome containing the previously identified QTLs for the same trait (Table 4) (Yu et al., 2006; Li et al., 2010, 2014; Deng et al., 2011; Guo et al., 2017).

The combination of GWAS and linkage mapping successfully addressed the constraints imposed by either method used separately. In this study, both GWAS and QTL mapping concurrently detected a single, reliable, colocalized QTL, *qTSE8* which likely contains important SER-associated genes. The identification and functional study of these genes is imperative to thoroughly decipher both the molecular and genetic foundation of SER. Transcriptomic analysis is another powerful method for mining genes associated with a given trait. Previously (Xia et al., 2020), we had performed a transcriptomic analysis of the two RIL parental lines, and upon comparing with the results of FPKM and qRT-PCR, we found 17 candidate genes showing significant differences in expression levels between the two parents at reliable interval. Additionally, 13 candidate genes were associated with significant differences in the SER among different haplotypes. Taken together, these results illustrate that combining association and linkage mapping with RNA-seq can be a robust approach to mine for target genes.

## Data availability statement

Publicly available datasets were analyzed in this study. This data can be found at: http://www.ncbi.nlm.nih.gov/bioproject/PRJNA260762; http://www.ncbi.nlm.nih.gov/bioproject/PRJNA609211.

## Author contributions

YL: writing—original draft, writing—review and editing, and funding acquisition. DF: resource and investigation. DK, AZ, and FW: investigation. XM, LW, HX, and GL: methodology. XY: supervision and funding acquisition. LL: conceptualization, supervision, and writing—review and editing. All authors contributed to the article and approved the submitted version.

## Funding

This work was supported by the Shanghai Municipal Commission of Science and Technology (21ZR1456800 and 19391900100) and Open Funding Project of the State Key Laboratory of Biocatalysis and Enzyme Engineering (SKLBEE2021023).

## Conflict of interest

The authors declare that the research was conducted in the absence of any commercial or financial relationships that could be construed as a potential conflict of interest.

## Publisher's note

All claims expressed in this article are solely those of the authors and do not necessarily represent those of their affiliated organizations, or those of the publisher, the editors and the reviewers. Any product that may be evaluated in this article, or claim that may be made by its manufacturer, is not guaranteed or endorsed by the publisher.
