# Supplementary figures and images for: Linkage mapping and association analysis to identify a reliable QTL for stigma exsertion rate in rice
Source: Front Plant Sci. 2022 Aug 23;13:982240. doi: 10.3389/fpls.2022.982240 (PMC9445662; doi:10.3389/fpls.2022.982240)

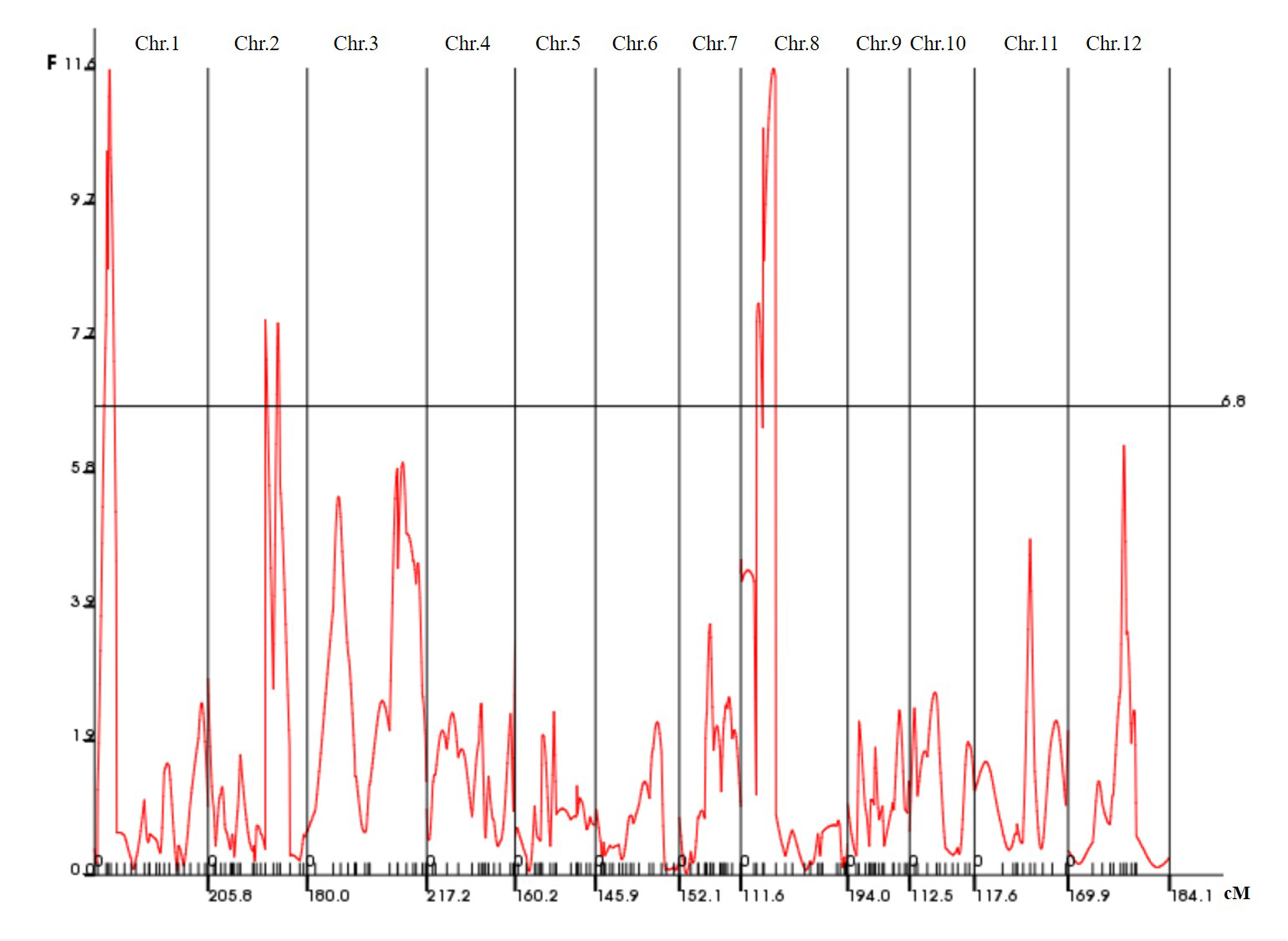

Supplement: Supplementary file 2 [file Image_1.jpg]

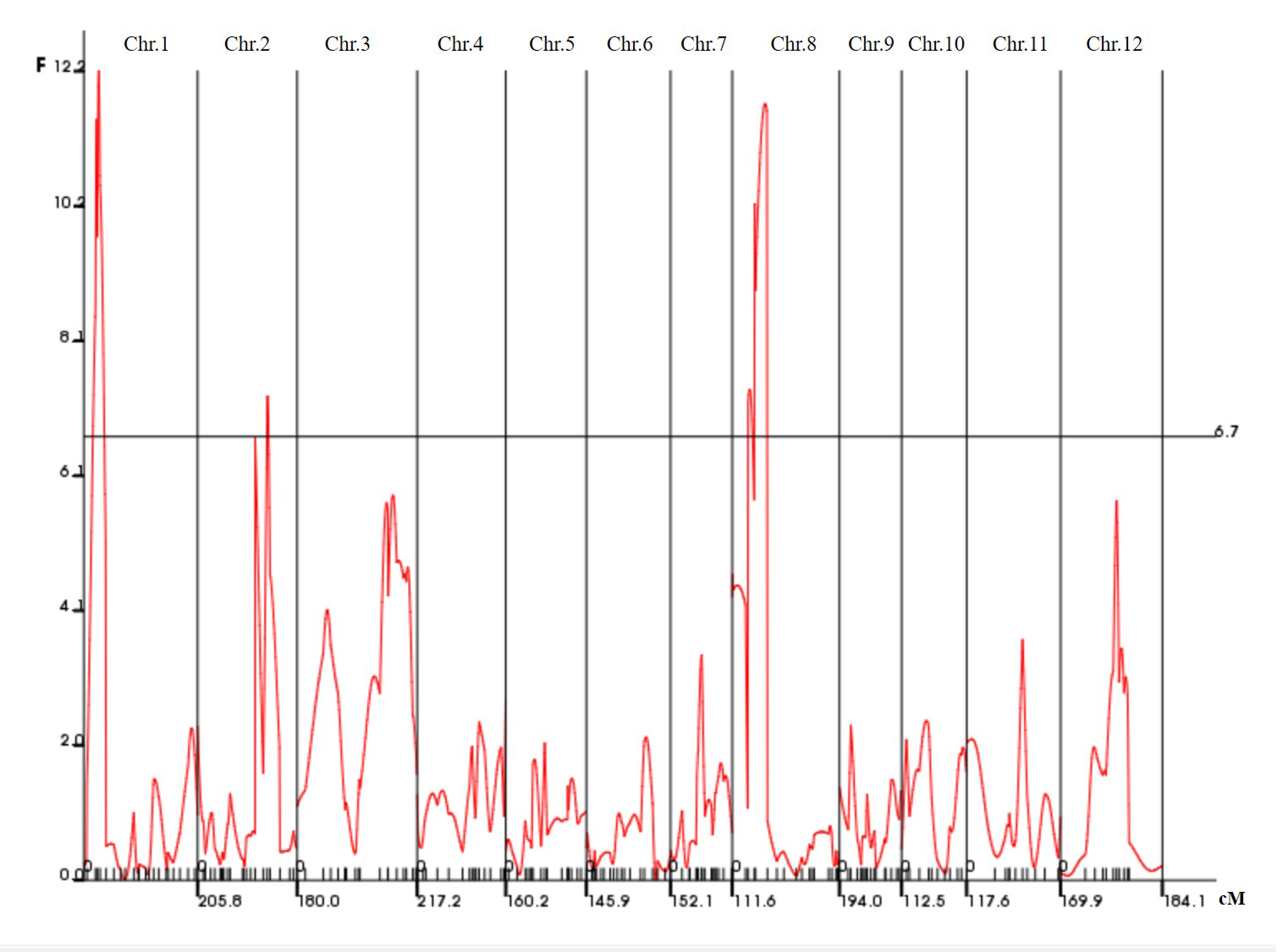

Supplement: Supplementary file 3 [file Image_2.jpg]

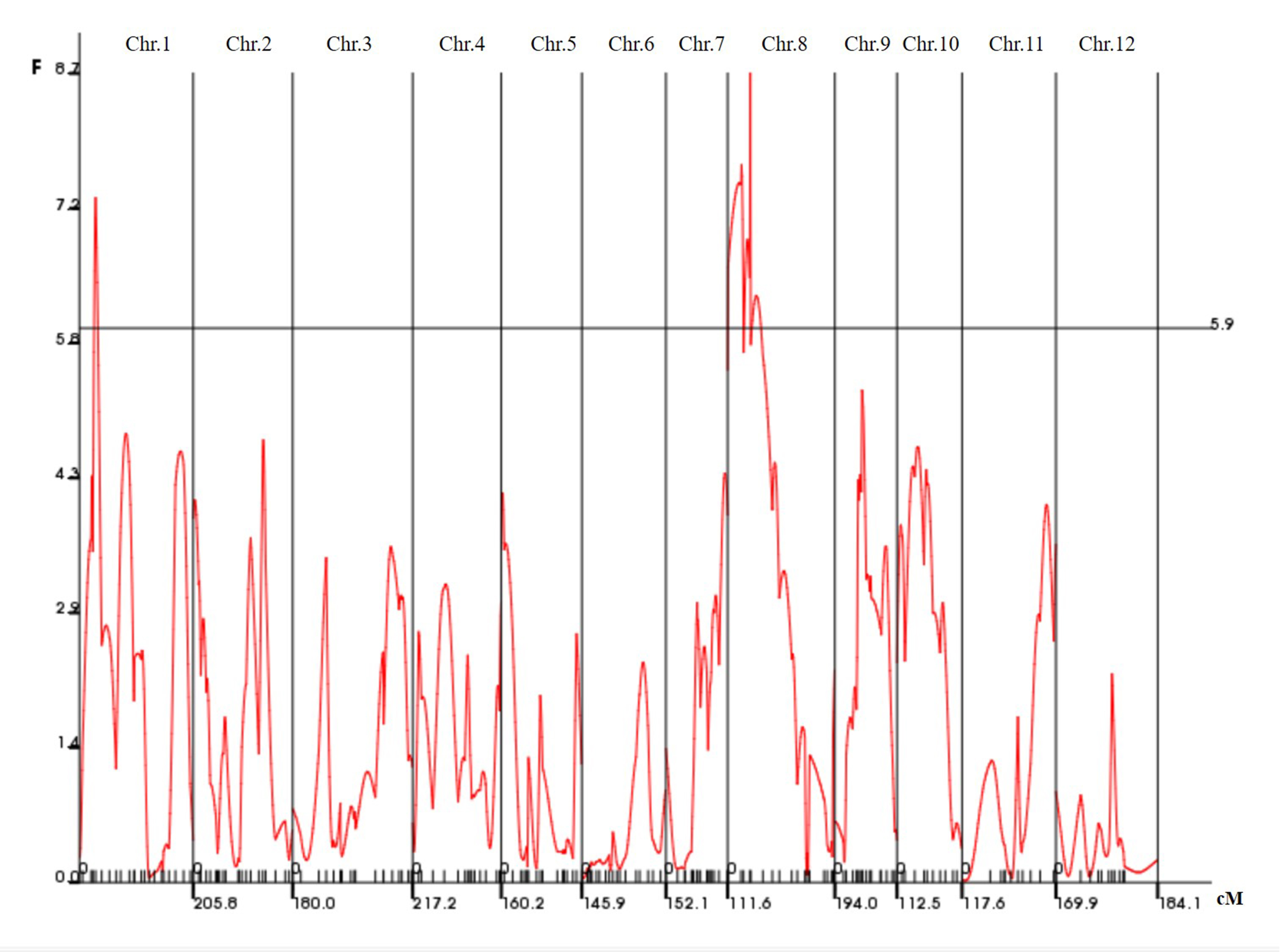

Supplement: Supplementary file 4 [file Image_3.jpg]
